# Supplementary material for: HKT1;1 and HKT1;2 Na+ Transporters from Solanum galapagense Play Different Roles in the Plant Na+ Distribution under Salinity
Source: Int J Mol Sci. 2022 May 4;23(9):5130. doi: 10.3390/ijms23095130 (PMC9103179; doi:10.3390/ijms23095130)
Supplement: Supplementary file 1 [file ijms-23-05130-s001.zip › ijms-1701531-supplementary.pdf]

International Journal of
Molecular Sciences

MDPI

Article

HKT1;1 and HKT1;2 Na^+ Transporters from *Solanum galapagense* Play Different Roles in the Plant Na^+ Distribution under Salinity

Maria J. Asins [1,*], Maria R. Romero-Aranda [2], Jesus Espinosa [3], Paloma González-Fernández [3], Emilio Jaime-Fernández [2], Jose A. Traverso [4], Emilio A. Carbonell [1] and Andres Belver [3]

1 Instituto Valenciano de Investigaciones Agrarias (IVIA), Moncada, 46113 Valencia, Spain; carbonell.emi@gmail.com
2 Department of Plant Breeding and Biotechnology, La Mayora Institute for Mediterranean and Subtropical Horticulture (IHSM), UMA/CSIC, Algarrobo-Costa, 29750 Malaga, Spain; rromero@eelm.csic.es (M.R.R.-A.); emilio@ihsm.uma-csic.es (E.J.-F.)
3 Department of Stress, Development and Signaling of Plants, Estación Experimental del Zaidín (EEZ), Consejo Superior de Investigaciones Científicas (CSIC), C/Prof. Albareda 1, 18008 Granada, Spain; jesusespinosa@eez.csic.es (J.E.); paloma.gonzalez.fernandez@hotmail.es (P.G.-F.); andres.belver@eez.csic.es (A.B.)
4 Department of Cellular Biology, Faculty of Sciences, University of Granada, 18071 Granada, Spain; traverso@ugr.es
* Correspondence: mjasins@ivia.es; Tel.: +34-96-342-4067

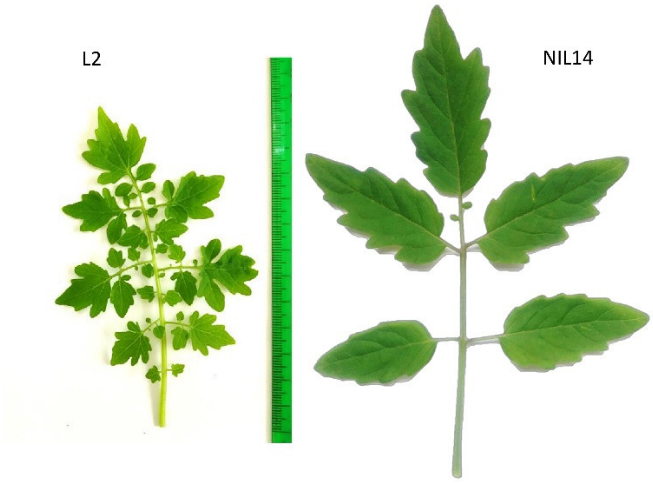

Figure S1. Mature fully expanded leaves of NIL14, from which silenced HKT1 lines were obtained, and of the salt tolerant line L2 [4] that was the wild parent of the RIL population from which NIL14 was obtained. L2 has now been reclassified as *S. galapagense* because of its leaf morphology [38–39].

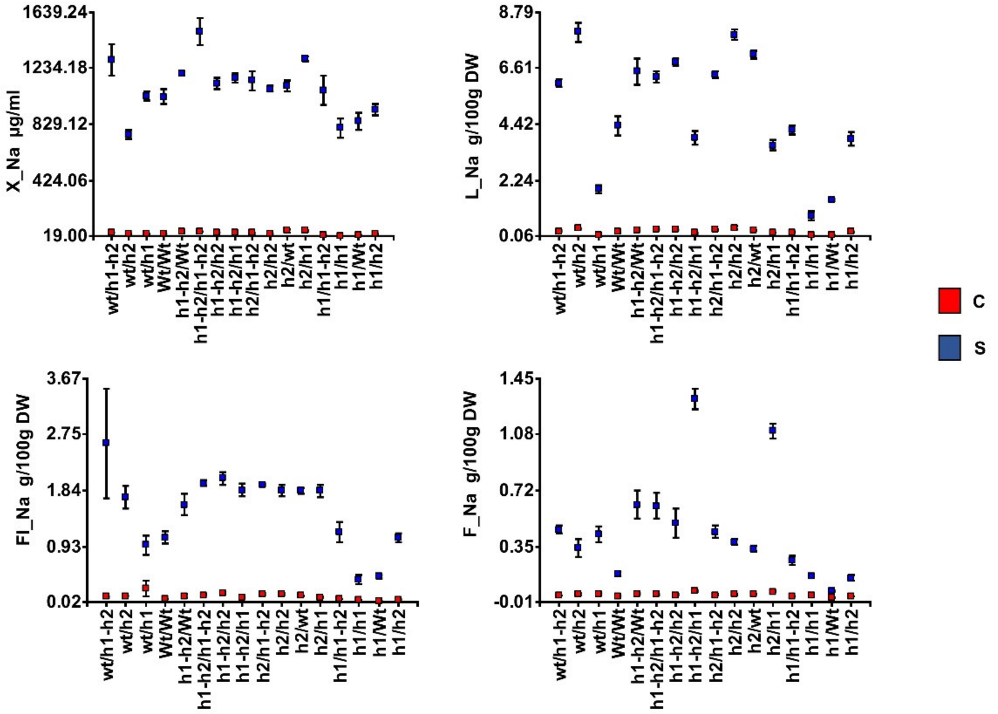

Figure S2. Means and standard errors for Na contents of leaf (L_Na), inflorescence (Fl_Na), Fruit (F_Na) and xylem sap Na concentration (X_Na) of the 16 reciprocal rootstock/scion graft combinations involving non-silenced (Wt), single RNAi-silenced lines for *SgHKT1;1* (h1), *SgHKT1;2* (h2), as well as doubly silenced line at both loci (h1–h2) (For instance, Wt/h1 indicates wild type rootstock and silenced *SgHKT1;1* scion genotypes). Red and blue squares correspond to control and salinity conditions, respectively.

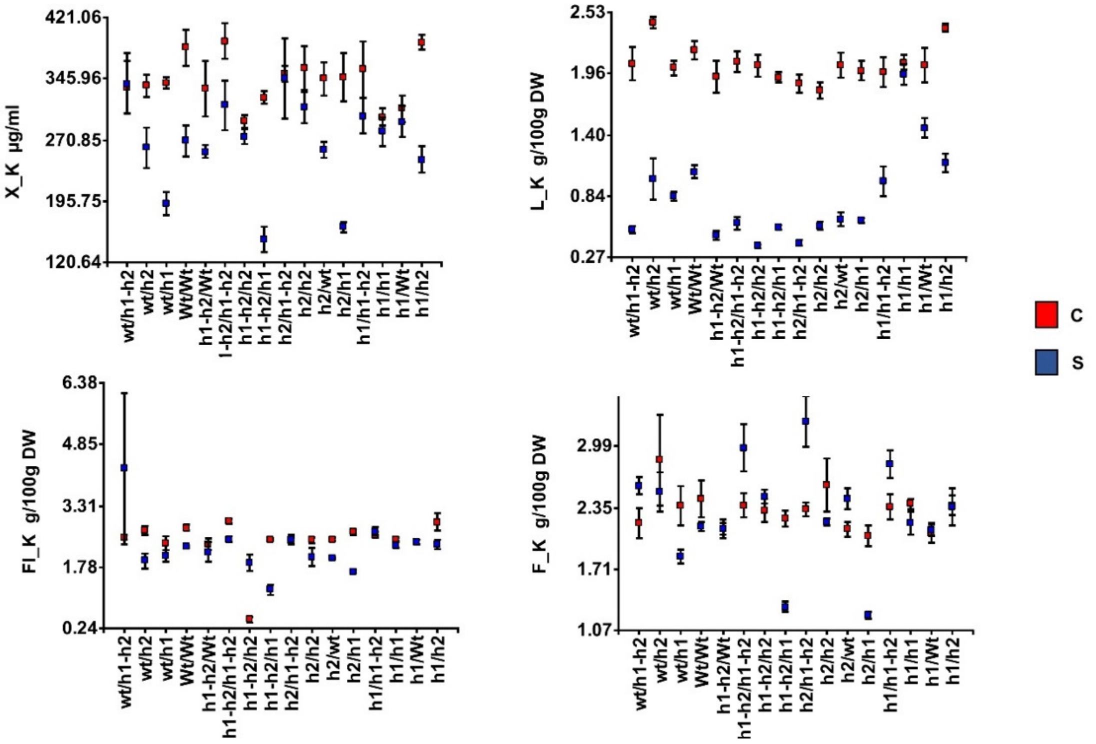

Figure S3. Means and standard errors for K contents of leaf (L_K), inflorescence (Fl_K), Fruit (F_K) and xylem sap K^+ concentration (X_K) of the 16 reciprocal rootstock/scion graft combinations involving non-silenced (Wt), single RNAi-silenced lines for *SgHKT1;1* (h1), *SgHKT1;2* (h2), as well as doubly silenced line at both loci (h1–h2). Red and blue squares correspond to control and salinity conditions, respectively.

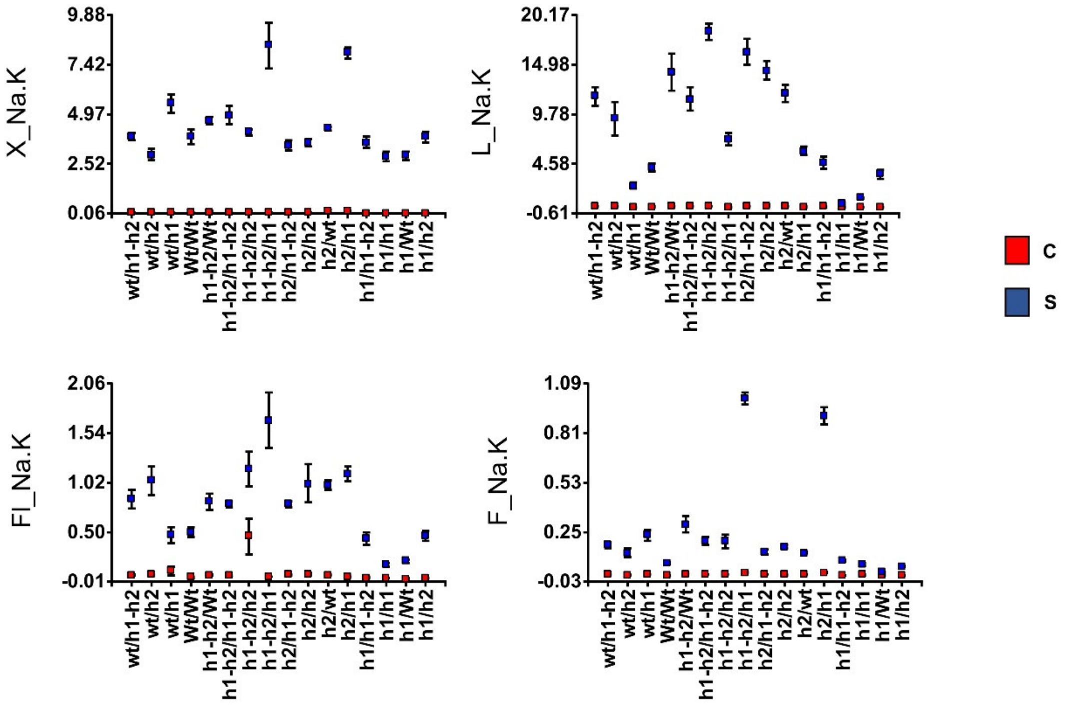

Figure S4. Means and standard errors for Na/K ratio of leaf (L_Na.K), inflorescence (Fl_Na.K), Fruit (F_Na.K) and xylem sap (X_Na.K) of the 16 reciprocal rootstock/scion graft combinations involving non-silenced (Wt), single RNAi-silenced lines for *SgHKT1;1* (h1), *SgHKT1;2* (h2), as well as doubly silenced line at both loci (h1-h2). Red and blue squares correspond to control and salinity conditions, respectively.

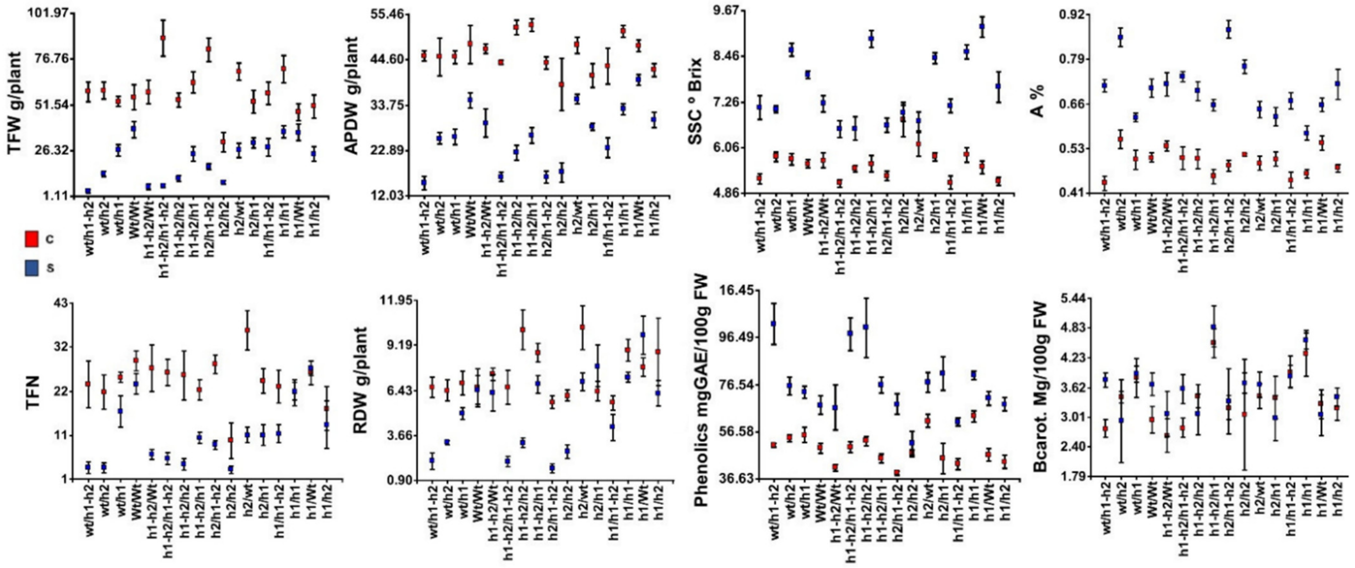

Figure S5. Means and standard errors for total fruit weight (TFW), total fruit number (TFN), dry weight of the aerial part of the plant (APDW), root dry weight (RDW) and fruit contents of soluble solids content (SSC), phenolics, β-carotenoids, and fruit juice acidity (A) of the 16 reciprocal rootstock/scion graft combinations involving non-silenced (Wt), single RNAi-silenced lines for *SgHKT1;1* (h1), *SgHKT1;2* (h2), as well as doubly silenced line at both loci (h1–h2). Red and blue squares correspond to control and salinity conditions, respectively.

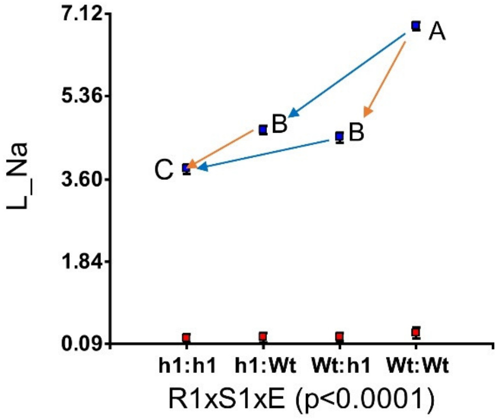

Figure S6. Adjusted means and standard errors of significant triple interaction R1 × S1 × E ($p < 0.0001$) for leaf Na content (L_Na, g/100g dry weight). Means of genotypic combinations under salinity (blue squares) with different letters were significant by LSD Fisher ($\alpha = 0.05$). No differences were found in control (red squares). Arrows show the directions of the effects of silencing *SgHKT1;1* at the scion (orange arrows) and at the root (blue arrows). In all cases, leaf Na content decreases.

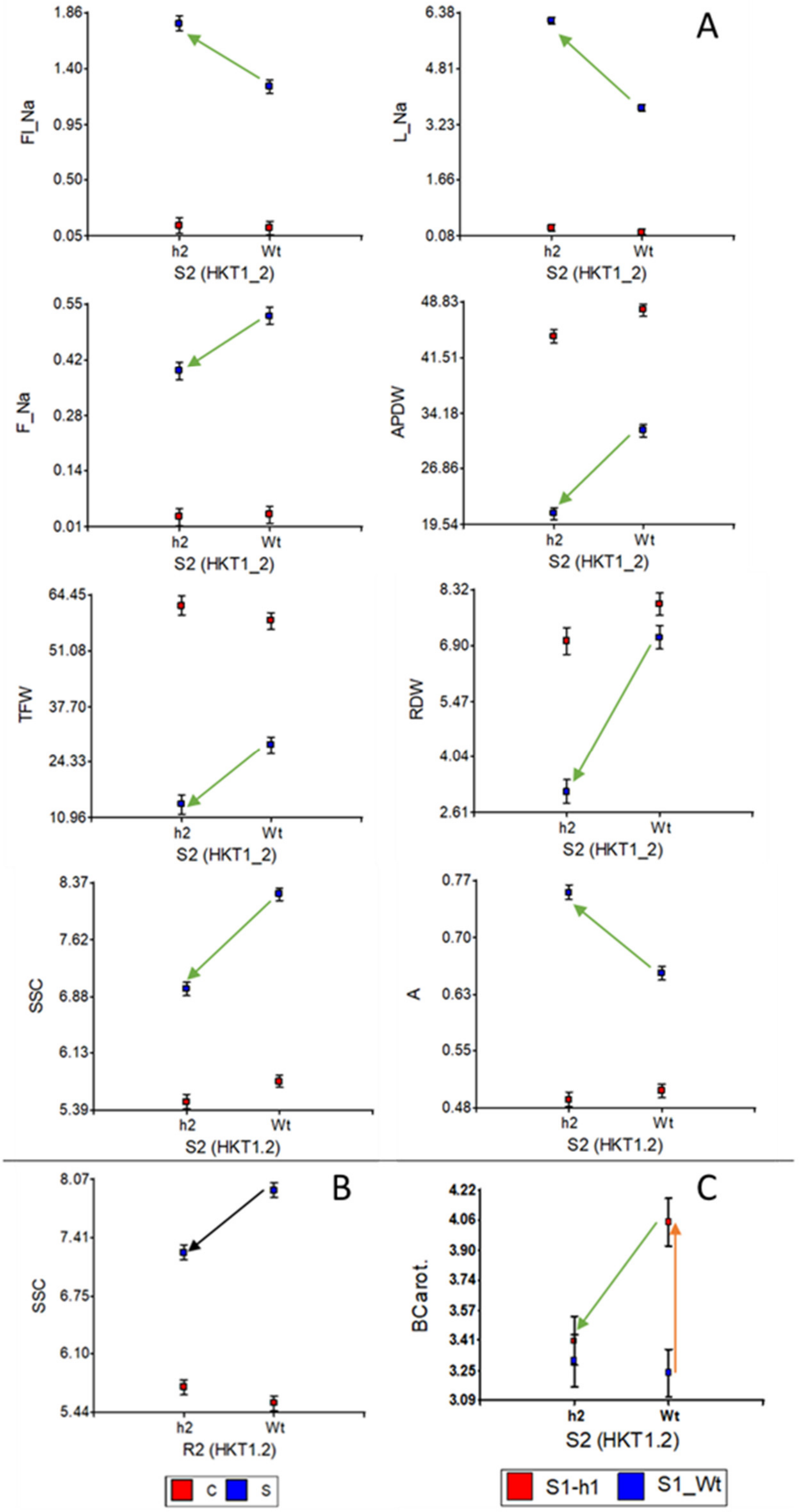

Figure S7. Adjusted means and standard errors of significant S2×E interactions (**A**) for Na contents of leaf, fruit and inflorescence, vegetative traits (APDW, RDW), and agronomic traits (TFW, SSC and A). Green arrows show the direction of the effect on the trait when silencing SgHKT1;2 at the scion. Red and blue squares correspond to control and salinity conditions, respectively. (**B**) Adjusted means and standard errors of significant R2 × E interaction for SSC, and (**C**) S2 × S1 for β-carotenoids. The black arrow shows the direction of the effect on SSC (decreasing SSC) when silencing SgHKT1;2 at the rootstock. The upwards orange arrow shows the increasing effect on β-carotenoids fruit content when silencing SgHKT1;1 at the scion.

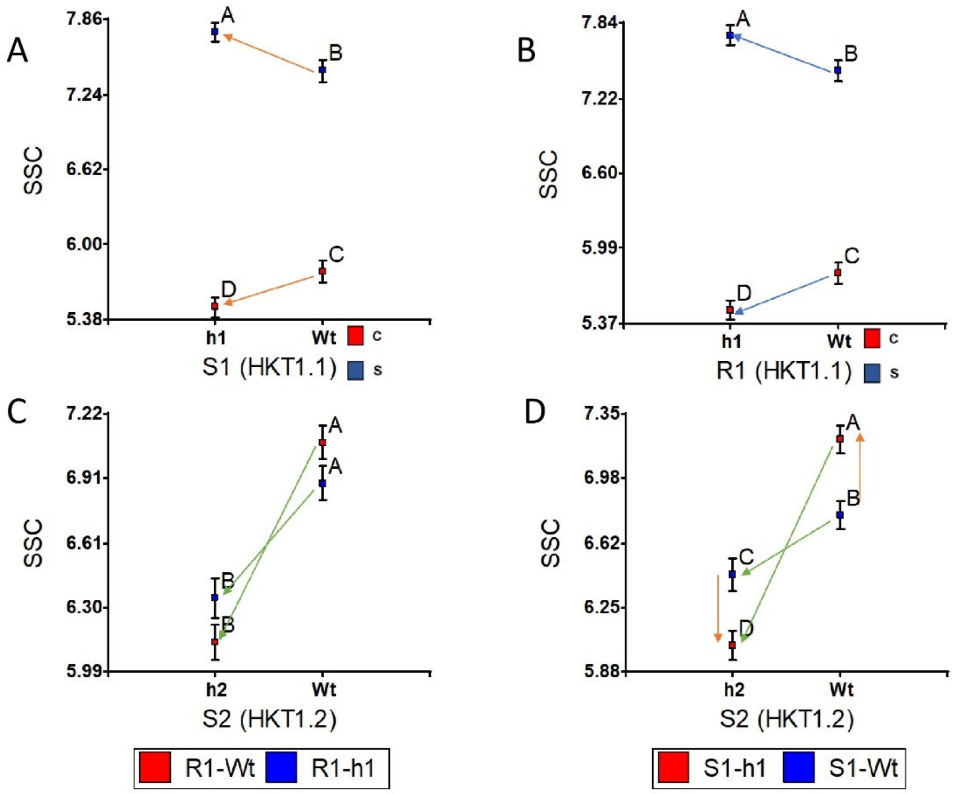

Figure S8. Adjusted means and standard errors of significant S1 × E (**A**), R1 × E (**B**), S2 × R1 (**C**) and S2 × S1 (**D**) interactions for SSC. Red and blue squares correspond to salinity and control conditions, respectively, in (**A**) and (**B**). Means with different letters are significantly different at 5% level. Arrows show the directions of the effects of silencing *SgHKT1;1* at the scion (orange arrows) and at the root (blue arrows), and silencing *SgHKT1;2* at the scion (green arrows).

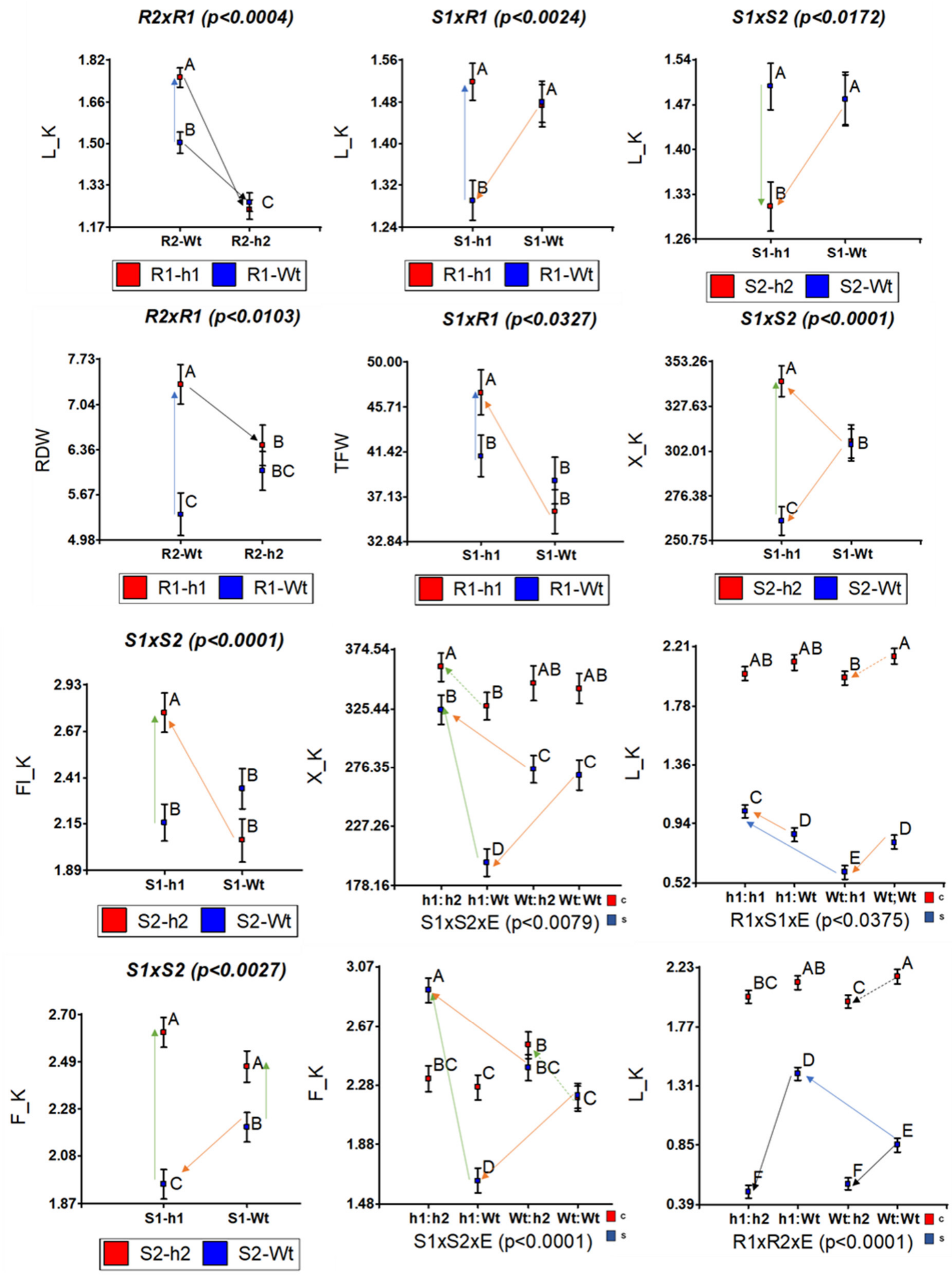

Figure S9. Adjusted means and standard errors of significant binary and triple interactions affecting K contents of leaf (L_K, g/100g DW), inflorescence (Fl_K, g/100g DW) and fruit (F_K, g/100g DW), and xylem sap [K+] (X_K, µg/ml). Two of these interactions R2 × R1 and R1 × S1 also affects fruit yield (TFW, g/plant), and R2 × R1 to root dry weight (RDW, g/plant). Arrows show the directions of the effects of silencing *SgHKT1;1* at the scion (orange arrows) and at the root (blue arrows), and silencing *SgHKT1;2* at the scion (green arrows) and at the root (black arrows). Arrows with a discontinuous line correspond to changes under control conditions. Means with different letters are significantly different at 5% level.

Table S1. Pearson coefficients of significantly correlated ($p < 0.05$) traits under control conditions (Pearson_C) and under salinity (Pearson_S). Shaded in yellow, Pearson coefficients for same trait, different salinity level.

Trait 1	Trait 2	Pearson_C	*p*–Value
βCarot.	TCarot.	0.94	<0.0001
APDW	RDW	0.67	0.0047
F_Na	F_Na.K	0.83	0.0001
F_Na	X_Na.K	0.5	0.0478
F_Na.K	X_Na	0.57	0.0212
F_Na.K	X_Na.K	0.6	0.0134
Fl_K	Fl_Na.K	−0.95	<0.0001
Fl_K	X_K	0.64	0.007
L_Na	βCarot.	−0.53	0.0366
L_Na	L_Na.K	0.97	<0.0001
L_Na.K	βCarot.	−0.52	0.038
Lyc.	βCarot.	0.53	0.0337
Lyc.	TCarot.	0.79	0.0003
RDW	AP.R	−0.88	<0.0001
SSC	Lyc.	0.6	0.0143
TFN	Lyc.	−0.53	0.0329
TFN	TFW	0.56	0.0231
TFN	X_Na.K	0.5	0.0464
TFW	Lyc.	−0.7	0.0027
X_K	APDW	−0.64	0.008
X_Na	X_Na.K	0.95	<0.0001
**Trait 1**	**Trait 2**	**Pearson_S**	***p*–value**
βCarot.	TCarot.	0.95	<0.0001
A	SSC	−0.61	0.0113
AP.R	A	0.81	0.0002
AP.R	SSC	−0.75	0.0009
APDW	A	−0.52	0.0388
APDW	AP.R	−0.75	0.0008
APDW	RDW	0.92	<0.0001
APDW	SSC	0.6	0.0132
F_K	A	0.59	0.0165
F_K	AP.R	0.82	0.0001
F_K	F_Na.K	−0.73	0.0015
F_K	Fl_K	0.53	0.0348
F_K	RDW	−0.67	0.0047
F_K	SSC	−0.75	0.0009
F_K	X_K	0.87	<0.0001
F_K	X_Na.K	−0.71	0.0021
F_Na	F_K	−0.54	0.0309
F_Na	F_Na.K	0.96	<0.0001
F_Na	Fl_Na	0.53	0.0358
F_Na	Fl_Na.K	0.8	0.0002
F_Na	L_K	−0.57	0.0207
F_Na	X_K	−0.62	0.0108
F_Na	X_Na	0.59	0.0159
F_Na	X_Na.K	0.92	<0.0001

F_Na.K	Fl_K	−0.51	0.0445
F_Na.K	Fl_Na.K	0.7	0.0023
F_Na.K	X_K	−0.76	0.0007
F_Na.K	X_Na.K	0.95	<0.0001
Fl_K	X_K	0.7	0.0027
Fl_Na	AP.R	0.53	0.0367
Fl_Na	APDW	−0.71	0.0022
Fl_Na	Fl_Na.K	0.77	0.0005
Fl_Na	L_K	−0.87	<0.0001
Fl_Na	L_Na	0.78	0.0004
Fl_Na	L_Na.K	0.8	0.0002
Fl_Na	RDW	−0.64	0.008
Fl_Na	SSC	−0.64	0.0071
Fl_Na	X_Na	0.68	0.004
Fl_Na.K	L_K	−0.74	0.001
Fl_Na.K	L_Na	0.58	0.0187
Fl_Na.K	L_Na.K	0.6	0.0142
Fl_Na.K	X_Na.K	0.62	0.0101
L_K	APDW	0.61	0.0123
L_K	L_Na.K	−0.81	0.0001
L_K	RDW	0.53	0.0352
L_K	SSC	0.57	0.022
L_K	X_Na	−0.72	0.0015
L_Na	A	0.71	0.002
L_Na	AP.R	0.61	0.012
L_Na	APDW	−0.53	0.0366
L_Na	L_K	−0.7	0.0025
L_Na	L_Na.K	0.86	<0.0001
L_Na	RDW	−0.66	0.0057
L_Na	SSC	−0.86	<0.0001
L_Na.K	A	0.59	0.0169
L_Na.K	AP.R	0.61	0.0116
L_Na.K	APDW	−0.61	0.0119
L_Na.K	RDW	−0.65	0.0062
L_Na.K	SSC	−0.8	0.0002
Lyc.	βCarot.	0.59	0.0153
Lyc.	TCarot.	0.81	0.0001
RDW	A	−0.67	0.0045
RDW	AP.R	−0.89	<0.0001
RDW	SSC	0.77	0.0005
TFN	A	−0.54	0.0319
TFN	AP.R	−0.54	0.0296
TFN	APDW	0.78	0.0004
TFN	Fl_Na	−0.86	<0.0001
TFN	Fl_Na.K	−0.67	0.0043
TFN	L_K	0.75	0.0009
TFN	L_Na	−0.83	0.0001
TFN	L_Na.K	−0.8	0.0002
TFN	RDW	0.75	0.0009
TFN	SSC	0.74	0.001
TFN	TFW	0.9	<0.0001

TFW	A	−0.56	0.0227
TFW	AP.R	−0.6	0.014
TFW	APDW	0.76	0.0006
TFW	Fl_Na	−0.77	0.0005
TFW	L_K	0.68	0.0035
TFW	L_Na	−0.76	0.0006
TFW	L_Na.K	−0.8	0.0002
TFW	RDW	0.75	0.0008
TFW	SSC	0.71	0.0022
X_K	AP.R	0.71	0.0022
X_K	RDW	−0.55	0.0258
X_K	SSC	−0.59	0.016
X_K	X_Na.K	−0.81	0.0002
X_Na	APDW	−0.55	0.0257
X_Na	X_Na.K	0.52	0.0377
**Trait_C**	**Trait_S**	**Pearson**	***p*–value**
βCarot._C	βCarot._S	0.66	0.0052
AP.R _C	AP.R _S	0.52	0.0399
F_Na.K_C	F_Na.K_S	0.89	<0.0001
F_Na_C	F_Na_S	0.89	<0.0001
L_Na.K_C	L_Na.K_S	0.88	<0.0001
L_Na_C	L_Na_S	0.97	<0.0001
TCarot._C	TCarot._S	0.53	0.0341
X_Na _C	X_Na _S	0.72	0.0017
X_Na.K_C	X_Na.K_S	0.56	0.025

Table S2. *p* values of significant effects and interactions of the Genotype (*S. galapagense* allele versus its silenced form) at HKT1.1 (1) and HKT1.2 (2), in the rootstock (R1 and R2), the scion, (S1 and S2), and the salinity treatment (E) for evaluated traits (see section *4.2* for trait abbreviations).

Trait	R2xS2	R2xS1	R1xS2	R1xS1	S1xS2	R1xR2	S2xE	S1xE	R2xE	R1xE	E	S2	S1	R2	R1
**F_Na**	<0.0001	0.0015			<0.0001	<0.0001	0.0022	<0.0001	<0.0001		<0.0001	0.0008	<0.0001	<0.0001	
**F_K**	0.0213			0.0370	0.0027		0.0001					<0.0001			
**F_Na/K**	<0.0001	<0.0001			<0.0001	0.0114	<0.0001	<0.0001	<0.0001		<0.0001	<0.0001	<0.0001	<0.0001	
**Fl_Na**	0.0007					<0.0001	<0.0001		<0.0001	0.0029	<0.0001	<0.0001		<0.0001	0.0002
**Fl_K**					0.0001		0.0053						0.0202	0.0004	
**Fl_Na/K**	0.0013				0.0001	<0.0001			<0.0001		<0.0001			<0.0001	
**L_Na**	0.0411	<0.0001	0.0377	<0.0001	0.0002	<0.0001	<0.0001	<0.0001	<0.0001	<0.0001	<0.0001	<0.0001	<0.0001	<0.0001	<0.0001
**L_K**				0.0024	0.0172	0.0004	0.0001		<0.0001	0.0019	<0.0001	0.0181		<0.0001	0.0046
**L_Na/K**		0.0001	0.0167	0.0287	0.0043	<0.0001	<0.0001	0.0004	<0.0001	0.0056	<0.0001	<0.0001	0.0013	<0.0001	0.0045
**X_Na**			0.0015		0.0010	0.0074		0.0003	<0.0001		<0.0001		<0.0001	<0.0001	
**X_K**	0.0279				<0.0001		0.0037				<0.0001	<0.0001			
**X_Na/K**					<0.0001		0.0001	0.0018	0.0048		<0.0001		0.0001	0.0083	
**TFN**			0.0392		0.0009		0.0460		0.0002		<0.0001	<0.0001		0.0150	
**TFW**		0.0236		0.0327	0.0480		<0.0001	0.0166	0.0002		<0.0001	0.0140	0.0014		
**APDW**							<0.0001	0.0011	0.0112		<0.0001	<0.0001	0.0001	0.0100	0.0008
**RDW**						0.0103	<0.0001		0.0372		<0.0001	<0.0001	0.0006		0.0002
**AR/R**	0.0065				0.0001	0.0301	<0.0001		0.0396			<0.0001	0.0059		0.0008
**A**		0.0004	0.0030				<0.0001			0.0454	<0.0001	<0.0001	0.0001		0.0059
**SSC**			0.0164		<0.0001		<0.0001	0.0003	<0.0001	0.0006	<0.0001	<0.0001		0.0024	
**βCarot.**				0.0008	0.0088							0.0333	0.0007		
**TCarot.**				0.0035	0.0232								0.0202		
**Phenolics**						0.0026					<0.0001				
